# Upper Extremity Muscle Weakness Following Axillary Botox Injection With Sterile Water Reconstitution: A Case Report

**DOI:** 10.1002/ccr3.71681

**Published:** 2025-12-17

**Authors:** Pouria Chaghamirzayi, Javad Karimi Rozveh, Mohammad Azizmanesh, Mohammad Reza Maghsoudi

**Affiliations:** ^1^ Clinical Research Development Unit of Shahid Madani Hospital Alborz University of Medical Sciences Karaj Iran; ^2^ Department of Emergency Medicine Alborz University of Medical Sciences Karaj Iran

**Keywords:** axillary, Botox, complications, muscle weakness, sterile water

## Abstract

Severe, unexpected pain during botulinum toxin injection is a critical red flag for reconstitution error. Using sterile water instead of normal saline may cause atypical, wider‐spread effects like proximal muscle weakness, even at standard doses. Strict adherence to saline reconstitution is essential to prevent this complication.

## 1 Introduction

Botulinum toxin is a neurotoxic protein derived from *Clostridium botulinum* , the bacterium responsible for botulism. In large amounts, the toxin can cause life‐threatening paralysis; however, in controlled doses, it has been widely adopted as a therapeutic and cosmetic agent. Since the 1970s, its applications have expanded beyond ophthalmology into neurology, dermatology, urology, and other fields [1, 2].

Primary axillary hyperhidrosis is a chronic autonomic disorder characterized by bilateral, excessive sweating of the axillae that surpasses thermoregulatory requirements [3]. The pathophysiology, while largely idiopathic, is strongly influenced by genetic factors, often demonstrating a pattern consistent with autosomal dominant inheritance with incomplete penetrance. The condition is fundamentally driven by dysregulation within the sympathetic nervous system, leading to the hyperstimulation of eccrine sweat glands [3]. Extensive clinical trials have established botulinum toxin as a highly effective and generally safe treatment for hyperhidrosis, providing rapid and long‐lasting reductions in excessive sweating and improving patients' quality of life [4]. For axillary hyperhidrosis, the standard dose typically ranges from 50 to 100 International Units (U) of botulinum toxin type A per axilla, a range well established as safe and effective [5, 6, 7, 8].

Of the seven botulinum toxin serotypes identified, only types A and B are used in clinical practice, with botulinum toxin type A being the most widely employed for both therapeutic and aesthetic purposes. The first FDA approval of onabotulinumtoxinA for glabellar frown lines occurred in 2002, followed by subsequent formulations and expanded indications approved in Europe and the United States in the following years [2].

Botulinum toxin injections are considered a safe procedure, with most adverse events limited to mild and localized reactions such as pain, swelling, erythema, bruising, or transient numbness [9]. Nevertheless, although rare, systemic complications may occur and be serious. Reported adverse outcomes include botulism‐like effects—manifesting as muscle weakness, dysphagia, dyspnea, or visual disturbances—as well as hypersensitivity reactions, anaphylaxis, and significant local infections [10]. Despite the widespread use and established safety profile of botulinum toxin, rare serious adverse effects such as iatrogenic muscle weakness remain underrecognized and are sparsely documented in the literature. Herein, we report a case of reversible proximal muscle weakness following botulinum toxin type A injection for cosmetic and therapeutic purposes, associated with improper reconstitution.

## 2 Case History/Examination

A 29‐year‐old woman (173 cm, 82 kg) was referred to our clinic with complaints of fatigue and proximal upper extremity weakness persisting for 6 weeks. She had no history of comorbidities or regular medication use, apart from occasional multivitamin intake. On initial evaluation, her vital signs were stable, and she was afebrile. Neurological examination revealed no focal deficits, cranial nerve abnormalities (specifically, no ptosis, diplopia, or dysphagia), and normal deep tendon reflexes. There were no muscle tenderness, atrophy, neck weakness, or respiratory complaints such as cough or dyspnea. The only abnormal finding was symmetric proximal muscle weakness in the upper extremities, graded 3+/5, though a more detailed assessment of individual muscle groups was not performed at the time. Muscle strength in all other extremities was normal and symmetrical.

## 3 Differential Diagnosis, Investigations, and Treatment

During the preceding 6 weeks, the patient had visited several clinics for severe fatigue and had received supportive treatments, including intravenous fluids and vitamin supplementation (B‐complex and vitamin C). These interventions alleviated her fatigue but did not affect her muscle weakness. Laboratory investigations performed during this period—including complete blood count (CBC), erythrocyte sedimentation rate (ESR), C‐reactive protein (CRP), and vitamin D levels—showed no evidence of inflammation or hematologic abnormality, except for vitamin D insufficiency (19.5 ng/mL).

Given her presentation, differential diagnoses included inflammatory or metabolic myopathies, muscular dystrophy, endocrine disorders, and neuromuscular junction disorders. Physical examination revealed no cutaneous manifestations suggestive of autoimmune or rheumatologic disease (e.g., dermatomyositis). The patient denied any family history of neuromuscular disorders or recent infections in the preceding 4 months.

Further history revealed that 2 weeks before symptom onset, the patient had undergone botulinum toxin type A (Siax) injections for cosmetic treatment of the upper face and axillary hyperhidrosis. The procedure was performed by reconstituting a 200‐unit vial with 4 mL of sterile water, resulting in a concentration of 5 units per 0.1 mL. A total of 180 units were injected: 20 units to the upper face and 80 units to each axilla. The axillary injections were administered subcutaneously as wheals (“blebs”) using a 30‐gauge needle, distributed over 16 sites per axilla (total volume of 1.6 mL per axilla). Unlike her previous sessions—performed every 6–8 months for the past 3 years by the same general practitioner at the same clinic, who had 6 years of experience in cosmetic injections—she reported experiencing unusually severe pain at the injection sites during this procedure (lasting for 2–4 h) and mild headache for 6 h post‐injections. The total dosage and injection pattern were identical to the regimen she had consistently and uneventfully received during all prior treatments. It was later discovered that sterile water, rather than normal saline, had been inadvertently used for reconstitution on this specific occasion, which was the first time this error had occurred and was presumed to account for her severe injection‐site pain.

We requested additional investigations, including endocrine function tests, electrolytes, muscle enzymes, inflammatory and rheumatologic markers, and infectious disease screening. All results were within normal limits (Table 1). Electromyography and nerve conduction studies (EMG‐NCV) were recommended, but the patient declined further evaluation. Given the absence of a confirmed diagnosis, the lack of progressive or life‐threatening manifestations, and the relatively mild clinical course, no pharmacologic therapy was initiated.

**TABLE 1:** The patient's laboratory findings.

Test category	Test name	Result	Unit
Hematology	WBC	7100	/μL
	RBC	4.66	10^6^/μL
	Hemoglobin (Hb)	14.3	g/dL
	Hematocrit (Hct)	42.9	%
	MCV	92.1	fL
	MCH	30.7	pg
	MCHC	33.3	g/dL
	Platelets	221,000	/μL
	RDW	12.5	%
Differential	Neutrophils	59%	%
	Lymphocytes	39%	%
	Monocytes	1%	%
	Eosinophils	1%	%
Biochemistry	Calcium (Ca)	9.6	mg/dL
	Phosphorus (Ph)	4.0	mg/dL
	Total Bilirubin	0.7	mg/dL
	Direct Bilirubin	0.2	mg/dL
	AST	38	U/L
	ALT	39	U/L
	Alk. P	191	U/L
	LDH	332	U/L
	CPK	186	U/L
	Aldolase	2.0	U/L
	BS	117	mg/dL
	Urea	24	mg/dL
	Creatinine	0.8	mg/dL
	Sodium (Na)	139.2	mmol/L
	Potassium (K)	4.25	mmol/L
	TSH	2.2	μU/mL
	CRP	1.3	mg/L
Serology	ANA	0.1 (Negative)	Titer
	RF (Qualitative)	Negative	
	ESR	11	mm/h

Abbreviations: Alk. P, Alkaline Phosphatase; ALT, Alanine Aminotransferase; ANA, Antinuclear Antibodies; AST, Aspartate Aminotransferase; BS, Blood Sugar; CPK, Creatine Phosphokinase; CRP, C‐Reactive Protein; ESR, Erythrocyte Sedimentation Rate; LDH, Lactate Dehydrogenase; MCH, Mean Corpuscular Hemoglobin; MCHC, Mean Corpuscular Hemoglobin Concentration; MCV, Mean Corpuscular Volume; RBC, Red Blood Cell count; RDW, Red Cell Distribution Width; RF, Rheumatoid Factor; TSH, Thyroid‐Stimulating Hormone; WBC, White Blood Cell count.

## 4 Conclusion and Results

Remarkably, approximately 20 days after her initial evaluation at our clinic (76 days after symptom onset, 88 days post‐Botox injection), the patient reported sudden and complete resolution of her fatigue and muscle weakness upon awakening in the morning. During this period, she had received no specific therapy other than a single weekly dose of vitamin D3 (50,000 IU). On re‐examination, muscle strength had fully recovered to 5′ 5 bilaterally.

Considering the temporal relationship, the exclusion of alternative etiologies, and the self‐limited course, a final diagnosis of botulinum toxin‐induced muscle weakness associated with improper reconstitution using sterile water was made. At 6 months of follow‐up, the patient remained asymptomatic, with no recurrence of fatigue or muscle weakness.

## 5 Discussion

Botulinum toxin type A is generally considered safe, with most adverse events limited to mild local reactions. Rarely, unwanted effects such as muscle weakness have been described. Our case demonstrates reversible proximal upper extremity weakness following injections where the toxin was inadvertently reconstituted with sterile water. The close temporal relationship, absence of alternative causes, and spontaneous recovery suggest a causal link. This case highlights that serious complications, while rare, can arise from a confluence of factors, including total dosage and critical errors in preparation, rather than a single cause.

A key factor in any botulinum toxin complication is the total dose administered. Our patient received 180 units (80 U per axilla), which is above the typical range of 50 U per axilla for hyperhidrosis but is not considered a high or toxic dose [5, 6, 7, 8]. The patient's own history substantiates this, as she had tolerated this exact dosage regimen without adverse effects during several previous sessions over 3 years. Furthermore, cases presenting with classic systemic botulism‐like symptoms, such as the one reported by Rouientan et al. [11], typically involve significantly higher doses, such as the 300‐unit overdose described. In contrast, Tay et al. [12] described a case of severe systemic botulism after only 100 units, but this was linked to an illicit product source and a non‐standard injection technique. Since the dosage was both tolerated by the patient and supported by clinical literature [5, 6, 7, 8], and the product was from a regulated source, the most plausible explanation for this adverse event is the documented procedural error.

Crucially, all other variables in our patient's treatment were consistent with her prior uneventful sessions. The injections were performed by the same general practitioner with 6 years of cosmetic injection experience, at the same clinic, using the same product (Siax) and the same injection sites and techniques. The only variable that changed was the reconstitution agent. This strongly implicates the use of sterile water as the primary novel causative factor in this adverse event.

The immediate and severe injection‐site pain experienced by our patient is a critical clue. This symptom is consistent with the findings of Swinnen et al. [13], where patients uniformly reported intense pain when botulinum toxin was reconstituted in sterile water. The most plausible explanation for this pain is not altered toxin diffusion, but the hypotonicity of sterile water. When injected into tissue, a hypotonic solution causes an osmotic imbalance, leading to cell swelling and disturbance, which is perceived as severe pain. Normal saline, being isotonic, avoids this effect and is therefore the standard and recommended diluent.

While the pain can be directly attributed to osmosis, the mechanism behind the subsequent proximal muscle weakness remains less clear but is likely multifactorial. This finding provides a mechanistic explanation for our observation. The study by Eisele et al. [14] demonstrated that botulinum toxin complexes dissociate instantaneously at neutral pH into the free, active neurotoxin, a process accelerated by saline. Consequently, upon injection, all formulations function identically as solutions of free toxin, regardless of their original complex size. This underscores that the variable in our case was not the toxin itself, but the diluent. We propose that reconstitution with sterile water—a hypotonic, non‐ionic solution—deviates from this intended pharmaceutical process, potentially altering the diffusion and uptake of the neurotoxin and leading to the atypical proximal weakness observed, rather than the classic cranial symptoms seen in overdose. We therefore hypothesize that the intense local tissue reaction and inflammation caused by the hypotonic solution, combined with this elevated but non‐toxic dose and a potentially altered neurotoxin pharmacokinetic profile, facilitated greater than expected systemic absorption or created an atypical tissue environment that predisposed to wider neuromuscular effects. This contrasts with the classic, dose‐dependent systemic toxicity seen in overdose cases [11, 12]. Instead, it suggests a scenario where a preparation error unmasked a latent risk associated with a higher‐than‐standard dose that was otherwise well tolerated under normal conditions.

In conclusion, this case underscores that serious complications can result from the interplay of multiple factors. Here, a dosage at the upper end of the therapeutic spectrum, while historically safe for the individual, became problematic only when combined with a significant reconstitution error. Therefore, the acute onset of severe, unexpected injection‐site pain should be recognized as a key clinical red flag for a reconstitution error and should prompt heightened monitoring for potential systemic neurological sequelae.

## Author Contributions

**Pouria Chaghamirzayi:** conceptualization, data curation, methodology, project administration, writing – original draft, writing – review and editing. **Javad Karimi Rozveh:** data curation, software, writing – original draft, writing – review and editing. **Mohammad Azizmanesh:** data curation, methodology, writing – original draft, writing – review and editing. **Mohammad Reza Maghsoudi:** data curation, methodology, project administration, supervision, writing – original draft, writing – review and editing.

## Funding

The authors have nothing to report.

## Ethics Statement

All authors and patients agreed to publish this article. Written informed consent was obtained from the patients to publish this case report and any accompanying data. A copy of the written permissions is available for review by the Editor‐in‐Chief of this journal. Ethical approval for this case report was not required as per the policies of the Ethics Committee of Alborz University of Medical Sciences. According to the institutional guidelines, case reports are considered exempt from ethical approval as they do not constitute research involving systematic investigation. This policy is in alignment with the guidelines of the Ethics Committee of Alborz University of Medical Sciences (IR.NREC.013.1395.12).

## Conflicts of Interest

The authors declare no conflicts of interest.

## Data Availability

All data generated or analyzed during this study are included in this published article (available).
